# Editorial: Socio-Emotional and Educational Variables in Developmental Language Disorder (DLD)

**DOI:** 10.3389/fpsyg.2022.882397

**Published:** 2022-03-25

**Authors:** Eva Aguilar-Mediavilla, Richard O'Kearney, Daniel Adrover-Roig, Gabriela Simon-Cereijido, Lucía Buil-Legaz

**Affiliations:** ^1^Investigation in Development, Education and Language Laboratory, Institute of Educational Research and Innovation, University of the Balearic Islands, Palma, Spain; ^2^Research School of Psychology, Australian National University, Canberra, ACT, Australia; ^3^Department of Communication Disorders, California State University, Los Angeles, CA, United States

**Keywords:** developmental language disorder (DLD), emotional development, education, social development, language development

Persons with Developmental Language Disorder (DLD) show a persistent language delay not resolved at age five that impacts everyday life communication and learning in the absence of a medical condition, such as brain injury, genetic conditions or chromosomal disorders, hearing loss, autism spectrum disorders or intellectual disability (Bishop et al., 2016, 2017). DLD not only affects a person's daily communication, but also many aspects related to it, such as social skills, emotional development, leadership, adaptive skills, and academic performance, which in turn can severely affect his/her quality of life (Durkin and Conti-Ramsden, 2010; Yew and O'Kearney, 2013; Aguilar-Mediavilla et al., 2019; Valera-Pozo et al., 2020).

The direction of the relationship between DLD and social-emotional and educational variables is not fully understood. Some studies have pointed out that socio-emotional variables and academic abilities could be either risk factors to DLD or offer protection against the impact of DLD (Conti-Ramsden and Durkin, 2016). Therefore, the interacting relations between socio-emotional and educational variables in DLD need to be further explored to disentangle those variables that are risk factors, those that could be protective factors, and those that are consequences of the DLD.

Although there is a genetic substrate for language disorders (Rice et al., 2020), we must not forget that an adequate environment can protect against this biological predisposition; this requires expanding interventions for DLD and taking into account social and environmental factors. In this sense, social variables significantly impact language acquisition and development at various levels. These variables can be fed back into each other to produce a circle of mutual influence. Thus, social, economic, family, and cultural differences are associated with variability in the characteristics of the quantity and quality of the linguistic inputs that contribute to language learning difficulties. These language difficulties affect children's acquisition of social and emotional skills, their self-esteem, and the quality of their social relationships, increasing the relative risk for social, cultural, family, and economic disadvantage.

In this way, although it is not easy to intervene because each family is unique and clinicians will not always be able to mitigate or modify the circumstances surrounding a particular child with DLD, it will be important to support families and provide the resources they may benefit from. It should be noted that while we have considered these social variables as potential risk factors, many of them can become protective factors depending on their access and implementation.

The current Research Topic of Frontiers in Psychology aims to explore the reciprocal and interconnected relationships between social, emotional, and education variables in children, adolescents, and adults with DLD. Hence, the works published in the present Special Issue (SI) have tried to integrate a comprehensive approach, including social, educational, and emotional differences, to provide an important framework to understand DLD.

Some studies in this Research Topic have focused on family-related variables (Aguilera et al.; Gough Kenyon et al.). Parental figures have an important role in children's linguistic skills with DLD. The mother's educational level, the family's support, and their socioeconomic status may greatly influence their children's performance in both oral language and reading outcomes. This finding highlights the fact that receiving adequate family support can enrich the linguistic environment and thus reduce the negative consequences on school performance. However, both family support and parental emotional regulation (ER) could be related to the emotional regulation and linguistic performance of their children. In this sense, one of the articles in this SI (Aguilera et al.) focuses on the essential core of emotional competence, referring to the human ability that allows modification of the quality, intensity, duration, and expression of emotions according to the goals that one intends to achieve (Gross, 2015). The results of this paper show that parental ER is an essential factor in explaining DLD children's ER capacity, particularly during childhood; accordingly, parents have a direct influence on the ER capacity of their children, in terms of both adaptive and non-adaptive strategies.

In addition, this Research Topic also highlights the importance of considering the perspectives of different agents (such as tutors or parents) together with the particular characteristics of the students and their perceptions, which might differ from those of other educational agents (Gough Kenyon et al.; Sureda-García et al.). When reported by others, social and emotional skills differ between children with DLD and their typically developing (TD) peers. Concretely, in the views of both peers and teachers, students with DLD have less social competence and suffer more victimization than their peers. This perceived social deficit may modulate their higher tendency to peer victimization, in conjunction with their language difficulties (Sureda-García et al.). In terms of self-reports, there are two aspects in which children with DLD have significantly lower scores than TD peers: empathic ability and positive mood. This finding could partially explain the social difficulties and the lower quality of peer relationships in children with DLD, together with their heightened negative affect in terms of anxious and depressive symptoms. As for wellbeing, adolescents with DLD tend to present a lower self-reported autonomy and worse relationships with their parents than their TD. Congruently, parents report that adolescents with DLD score lower than TD on all dimensions, namely physical and psychological wellbeing, autonomy and parent relations, social support and peers, and school environment (Gough Kenyon et al.). In consequence, the perceptions of parents on children's wellbeing may not align well with their own self-perceptions.

In the present compilation of articles, it is worth pointing out findings related to language, given their close relationship with ER. In this sense, the work by Aguilera et al. emphasizes the crucial role of vocabulary on the differences in ER between children with DLD and TD. Their work shows that, expressive vocabulary can modulate and help to develop adaptive ER strategies. This indicates that having a broad expressive vocabulary at school age is of great help for the development of the capacity to clarify, understand, regulate, and express emotions adequately at later stages, specifically in children with DLD (Aguilera et al.). Finally, difficulties in ER and in the ability to take the other's perspective (theory of mind and social cognition) among children and adolescents with DLD might impact their narrative skills. Broc et al. show that adolescents with DLD experience problems with coherence (respect for the narrative schema) when narrating a personal experience, but not in cohesion (anaphora and connectors) nor in social-cognitive skills. Thus, problems with coherence in the narratives of adolescents with DLD, which are not shown by students with high functioning autism, are related to their challenges in accessing the space-time distance necessary to account for a personal experience. This becomes more evident and exacerbated by the putative deficits in expressive language in people with DLD, which complicates the task of building arguments to provide coherence to their narratives.

In terms of reading processes, numerous studies with English-speaking children with DLD have reported that they do not learn how to read at a typical pace. Because not all languages present the same transparency level in their spelling systems, it is important to investigate atypical literacy development in languages such as Spanish, which is considered to have a transparent orthography. Lara-Diaz et al. indicate that Spanish-speaking children with DLD also exhibit deficits in phonological awareness and reading abilities, which are compounded by general academic delays. In their study, maternal education, rather than income level, appeared to be a protective factor against academic difficulties. The authors conclude that exploring the associations between extrinsic and intrinsic factors with oral language and reading in populations outside wealthy nations adds insights to the clinical implications for both local practitioners and practitioners in rich countries working with immigrant families.

In view of these findings, we need to broaden the focus of research on the population with DLD, especially in children and adolescents, to include families as an essential element of assessment and intervention, which must be understood as a comprehensive process. In addition, the studies included in the present SI also highlight the need to consider the psychological wellbeing of children and adolescents with DLD, given their clinical correlates. The evidence from the studies support the growing awareness that DLD is an important risk factor for the development of enduring emotional and behavioral difficulties, which not only cascade to other developmental problems in educational attainment, peer relationships and self-construct but also interfere with the trajectory of language development itself. The work described in these studies also reinforces the need for improving the knowledge of the mechanisms through which this risk is transmitted in order to improve the effectiveness of preventive programs both for children with DLD and those with secondary language disorders associated with a biological condition, such as high functioning Autism Spectrum Disorder (ASD). This is currently a major challenge for existing mental health strategies and treatments for children and adolescents.

There are many specific implications that can be drawn from these studies. Firstly, regarding language skills, it has become clear again that the difficulties of children with DLD go beyond childhood and continue into adolescence, appearing in many other related areas of their lives. It is thus fundamental to implement direct actions that support the continuity and success of this population in educational contexts. Therefore, focusing on early intervention in different components of oral language and ecological language situations is mandatory in order to improve the development of other dimensions of language, such as reading or narrative skills, so that children with DLD can function optimally in a given socio-emotional context. Additionally, regarding the psychological implications, it is essential to point out that children and adolescents with DLD tend to be more victimized than their peers. It is vital to implement training programs in schools around different prosocial skills such as conflict resolution, anger management, the ability to forgive others, and even general social skills. In this sense, we should consider both their perceptions using self-reported assessment tools of victimization, and the information obtained from their peers (sociogram), as well as scales reported by other agents, such as teachers and families.

## Author Contributions

EA-M and LB-L wrote the first draft of this editorial. RO'K, DA-R, and GS-C edited consecutive versions. The overall Research Topic has been conceptualized by EA-M, RO'K, DA-R, GS-C, and LB-L. All authors have agreed on the final version.

## Funding

This work was supported by the Grant EDU2017-85909-P funded by MCIN/AEI/10.13039/501100011033 and by ERDF A way of making Europe. The funding sources had no involvement in the study design, analysis and interpretation of data, and writing of the report or submission for publication.

## Conflict of Interest

The authors declare that the research was conducted in the absence of any commercial or financial relationships that could be construed as a potential conflict of interest.

## Publisher's Note

All claims expressed in this article are solely those of the authors and do not necessarily represent those of their affiliated organizations, or those of the publisher, the editors and the reviewers. Any product that may be evaluated in this article, or claim that may be made by its manufacturer, is not guaranteed or endorsed by the publisher.
